# Linfedema e febre chicungunha

**DOI:** 10.1590/1677-5449.003717

**Published:** 2017

**Authors:** Marcos Arêas Marques, Arno Von Ristow

**Affiliations:** 1 Universidade do Estado do Rio de Janeiro – UERJ, Rio de Janeiro, RJ, Brasil.; 2 Pontifícia Universidade Católica do Rio de Janeiro – PUC-Rio, Rio de Janeiro, RJ, Brasil.

No relato de caso da edição anterior “Trombose venosa profunda e vírus chicungunha”^1^, os autores chamam atenção para a possibilidade da ocorrência da trombose venosa profunda de membros inferiores (MMII) como uma complicação vascular aguda, de origem multifatorial, da febre chicungunha (FC). Porém, o paciente apresentado no relato permaneceu com edema volumoso de MMII, mesmo após os 90 dias de tratamento anticoagulante com apixabana, e houve comprovação da recanalização com refluxo leve de veia poplítea direita ao eco Doppler colorido (EDC). As alterações do EDC não justificariam o edema volumoso e bilateral dos MMII e, além disto, o edema era clinicamente compatível com edema de origem linfática. Diante desses fatos, foi solicitada uma linfocintilografia de MMII para complementação do diagnóstico clínico e orientação da terapia física complexa.

**Figura 1:**
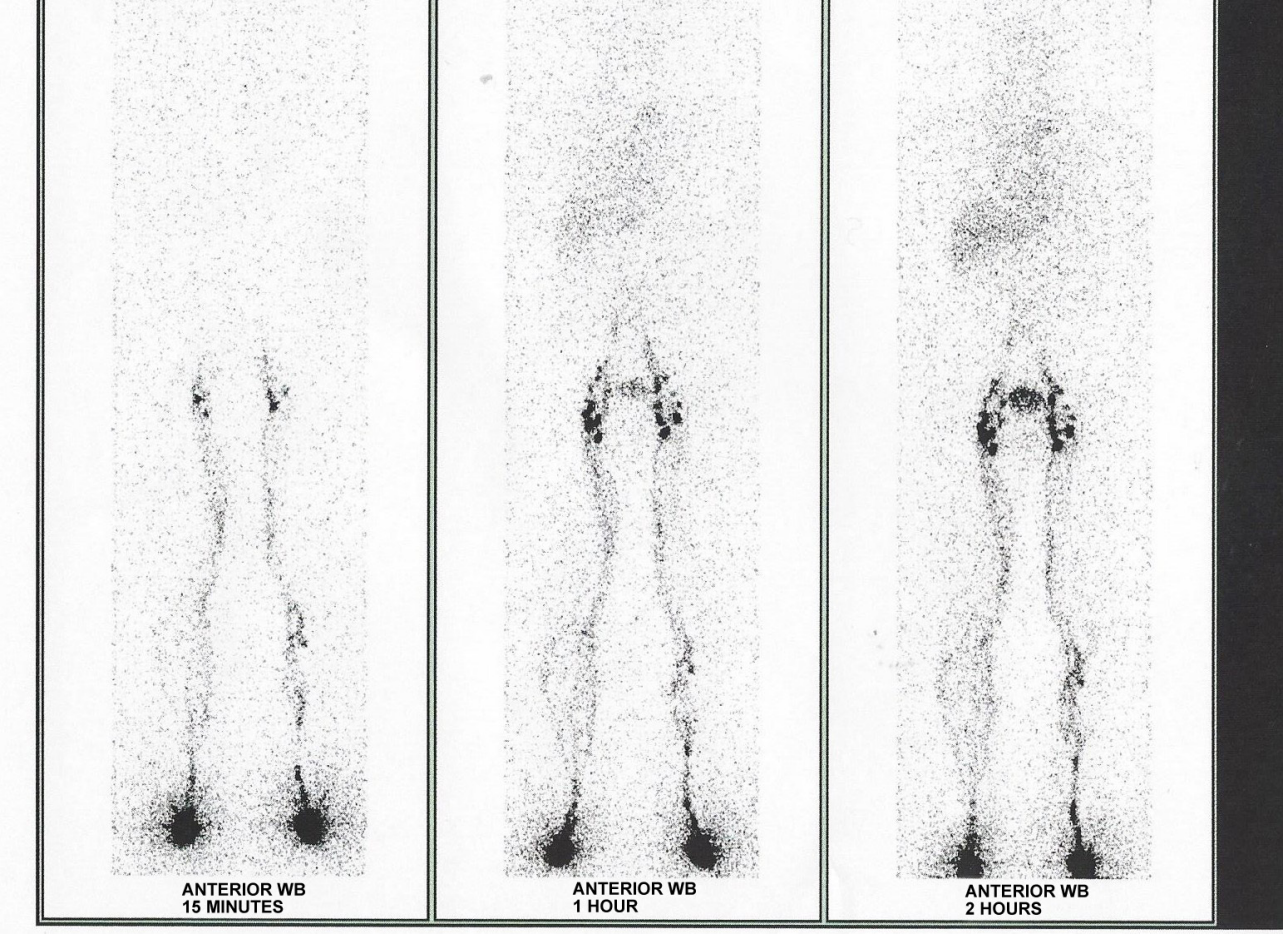
Linfocintilografia de membros inferiores (WB: *whole body*, ou corpo todo).

O exame (Figura 1) mostra alterações como refluxo dérmico, varicosidades linfáticas e padrão de hiperfluxo, que corroboram o diagnóstico clínico.

Como foi introduzida apenas recentemente no continente americano (2013), a FC e suas complicações vasculares ainda estão sendo estudadas e não há dados na literatura que comprovem essas complicações. Porém, uma recente tese de mestrado da Universidade Federal de Pernambuco (UFPE), intitulada: “Febre Chikungunya e linfedema de membros inferiores: comprovação linfocintilográfica”, um estudo observacional, prospectivo no qual pacientes na fase aguda ou subaguda da FC que evoluíram para edema de MMII foram submetidos a avaliação clínica e a linfocintilografia no início do estudo e após 90 dias, documenta as anormalidades da drenagem linfática de MMII causadas pela FC em mais da metade dos 32 pacientes avaliados.

Portanto, cremos que devemos ficar cada vez mais atentos às possíveis complicações vasculares que podem estar associadas à FC.
