# Supplementary material for: Linfedema e febre chicungunha
Source: J Vasc Bras. 2017 Apr-Jun;16(2):178–9. [Article in Portuguese] doi: 10.1590/1677-5449.003717 (PMC5915867; doi:10.1590/1677-5449.003717)
Supplement: Translation

# Translation: Lymphedema and Chikungunya fever

**Authors:** Marcos Arêas Marques, Arno Von Ristow

**Affiliations:** 1 Universidade do Estado do Rio de Janeiro – UERJ, Rio de Janeiro, RJ, Brazil.; 2 Pontifícia Universidade Católica do Rio de Janeiro – PUC-Rio, Rio de Janeiro, RJ, Brazil.

In a case report published in the last issue of this journal, entitled “Deep venous thrombosis and chikungunya virus”,^1^ the authors draw attention to the possibility of lower limb deep venous thrombosis as an acute vascular complication of chikungunya fever (CF), with multifactorial origins. However, the patient described in the report still had large volume edema of the lower limbs after 90 days on anticoagulant treatment with apixaban and color Doppler ultrasonography confirmed recanalization of the right popliteal vein with mild reflux. Doppler ultrasound findings did not explain the bilateral large volume edema of the lower limbs and the edema was also clinically compatible with edema of lymphatic origin. In view of this, lower limb lymphoscintigraphy was conducted to supplement the clinical diagnosis and help guide complex physiotherapy.

Lymphoscintigraphy (Figure 1) showed abnormalities such as dermal backflow, lymphatic varicosities and overflow pattern, confirming the clinical diagnosis.

**Figure 1:** Lower limb lymphoscintigraphy (WB: whole body).

Since CF was only recently introduced to the Americas (2013), its vascular complications are still being studied and there are no published data that demonstrate these complications. However, a recent Masters dissertation was presented to the Universidade Federal de Pernambuco (UFPE), entitled “Chikungunya Fever and lower limb lymphedema: lymphoscintigraphic proof” (“Febre Chikungunya e linfedema de membros inferiores: comprovação linfocintilográfica”). This dissertation was based on a prospective observational study of patients in the acute or subacute phase of CF who developed lower limb edema and underwent clinical assessment and lymphoscintigraphy at study outset and after 90 days and documented abnormalities of lower limb lymphatic drainage caused by CF in more than half of the 32 patients assessed.

We therefore believe that it is necessary to increase awareness of the possible vascular complications that may be associated with CF.
